# Broken Instrument Removal Methods with a Minireview of the Literature

**DOI:** 10.1155/2024/9665987

**Published:** 2024-06-13

**Authors:** Mohsen Aminsobhani, Nasim Hashemi, Fatemeh Hamidzadeh, Pegah Sarraf

**Affiliations:** ^1^ Department of Endodontics School of Dentistry Tehran University of Medical Sciences, Tehran, Iran; ^2^ School of Dentistry AJA University of Medical Sciences, Tehran, Iran

## Abstract

Instrument fracture in the root canal system is an unpleasant incident that may occur during root canal treatment. Comprehensive cleaning of the root canal system is often impossible in the presence of a broken instrument. Therefore, it is often imperative to remove the broken fragment from the root canal system. To date, various methods have been proposed for the removal of broken instruments from the root canal system. However, no consensus has been reached on a safe technique with a high success rate for broken instrument removal. This case series reports six cases of successful removal of broken instruments using different methods including the ultrasonic, tube-and-glue, tube-and-wire, tube-and-internal shaft, and the forceps techniques and also provides a brief review of the relevant literature.

## 1. Introduction

Instrument fracture is a common occurrence during root canal treatment [1]. The broken fragment can obstruct the root canal system; complicate irrigation, disinfection, and cleaning and shaping of the root canal system; and compromise the outcome, especially in infected teeth. Thus, prevention of instrument fracture is crucial and should take precedence over retrieval [2, 3].

Hand and rotary instruments made of stainless steel and nickel-titanium (NiTi) are widely employed for cleaning and shaping of the root canal system due to their flexibility and resistance to torsional fracture. They enable achieving predictable results with nonsurgical endodontic treatment [4]. However, instrument fracture can still occur despite adherence to the recommended protocols [5].

There are two approaches for the nonsurgical management of a broken instrument in the root canal system: fragment removal and fragment bypass. Optimally, the broken instrument should be removed from the root canal to facilitate effective cleaning and shaping [6]. If nonsurgical techniques fail to remove broken instruments, surgical approaches including apicoectomy, root amputation, or intentional replantation can be used. Intentional replantation (IR) refers to the deliberate extraction and subsequent reinsertion of an endodontically treated tooth into its socket with the aim of correcting an evident clinical or radiographic endodontic failure. This method is recommended in cases where an instrument is lodged in the canal and cannot be removed with nonsurgical techniques, when a posttype crown restoration necessitates retreatment, or when performing apical surgery would result in excessive bone loss leading and a risk of damage to vital structures [7, 8]. Removing a broken instrument from the root canal system is complicated and requires specialized training and experience, as well as a deep understanding of the available methods, techniques, and equipment. The success of the removal procedure depends on several factors including the location, visibility, size, length, and type of the fractured instrument, as well as the curvature and radius of the root canal. Experience and fatigue of the operator are also important factors that can affect the outcome [9, 10].

Many devices, techniques, and methods are available for the broken instrument retrieval [7]. In almost all cases, the retrieval procedure consists of two main phases: initially, the root canal is prepared using rotary or ultrasonic instruments to dislodge and loosen the broken fragment and expose the coronal portion for the subsequent step. Next, specialized devices or ultrasonic techniques are employed to remove the separated instrument [11].

Application of ultrasonic tips is an effective method for broken instrument removal from the root canal and is utilized in almost all retrieval procedures with the success rate varying from 33% to 100% [10]. However, even if the broken fragment in the canal becomes loose and mobile, the removal phase could be challenging. On the other hand, in some cases, the broken fragment remains immobile, and excessive ultrasonic use can result in excessive dentin removal, increasing the risk of iatrogenic errors such as transportation and perforation. Therefore, other methods should be considered for instrument removal [12–14].

Various methods have been devised for broken instrument retrieval. Application of these methods together with the use of a dental microscope may enable successful removal of the broken instrument with minimal dentin removal [11].

This case series reports six cases of successful removal of broken instruments using different methods and also provides a brief review of the relevant literature.

## 2. Case Presentation

Informed consent was obtained from all six patients concerning the utilization of their photographs and radiographs for inclusion in scientific publications and presentations without disclosing their identities.

### 2.1. Case 1: Removal of a Broken Instrument Using an Ultrasonic Instrument

**Figure 1:**
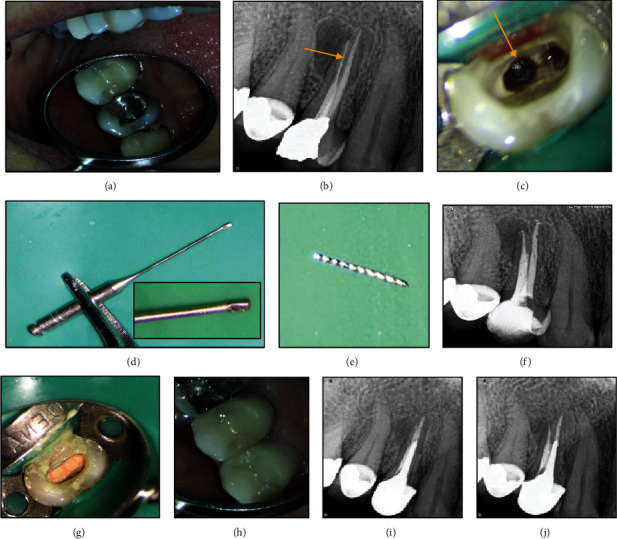
(a) Intraoral examination. (b) Radiographic examination showing periapical radiolucency and a broken instrument in the palatal canal. (c) Coronal portion of the broken file in the palatal canal was visualized under a DOM. (d) Modified Gates-Glidden drill. (e) Retrieved file fragment. (f, g) Canal preparation and obturation. (h, i) 6-month follow-up. (j) 2-year follow-up: evidence of healing of the apical radiolucency was seen.

A systemically healthy 30-year-old female patient was referred to an endodontist complaining of pain in her maxillary right first premolar while chewing. Clinical examination revealed that the tooth was sensitive to percussion and palpation, and had no mobility. The probing depth was within the normal range. Periapical radiograph revealed a broken instrument in the palatal canal and a radiolucency in the periradicular area of the palatal root (Figure 1(b)). Informed consent was taken from the patient. The amalgam restoration was removed, and endodontic retreatment was commenced by gutta-percha removal using rotary NiTi instruments and chloroform (Merck, Darmstadt, Germany) as gutta-percha solvent. The coronal portion of the broken instrument was visualized under a dental operating microscope (DOM; Magna Labomed, Labo America Inc., CA, USA) at ×4 magnification (Figure 1(c)). The tip of a #2 Gates-Glidden drill was cut by a carbide bur at high speed to prepare a staging platform (Figure 1(d)) in order to establish a straight-line access to the coronal part of the broken instrument with minimal dentin removal. Then, the dentinal walls around the coronal 2 mm of the broken instrument were removed using a size 25 U-file on a U-tip (NSK, Nakanishi, Japan) at ×15 magnification. The U-file moved around the broken instrument in a counterclockwise direction. After about 10 minutes, the broken file was successfully removed (Figure 1(e)). The canals were prepared with rotary NiTi instruments (DENCO Super Files III; Longhua, Shenzhen, China), irrigated with 5.25% hypochlorite solution and its activation with ultrasonic, and finally, rinsed with 17% EDTA. Subsequently, canals were obturated with gutta-percha points and AH 26 resin sealer (Dentsply-Tulsa Dental, Johnson City, TN, USA) using the cold lateral compaction technique (Figures 1(f) and 1(g)). The patient was then referred for coronal restoration. At the 2-year follow-up, the patient was completely symptom-free, and the healing of the apical radiolucency was evident in the periapical radiograph (Figure 1(j)).

### 2.2. Case 2: Removal of a Broken Instrument Using the Tube-and-Glue Technique

A 62-year-old male patient was referred to an endodontist due to an instrument fracture during root canal treatment of his maxillary left lateral incisor. The tooth was asymptomatic, with normal periodontal and periradicular status. Periapical radiograph showed a broken instrument in the middle third of the canal.

**Figure 2:**
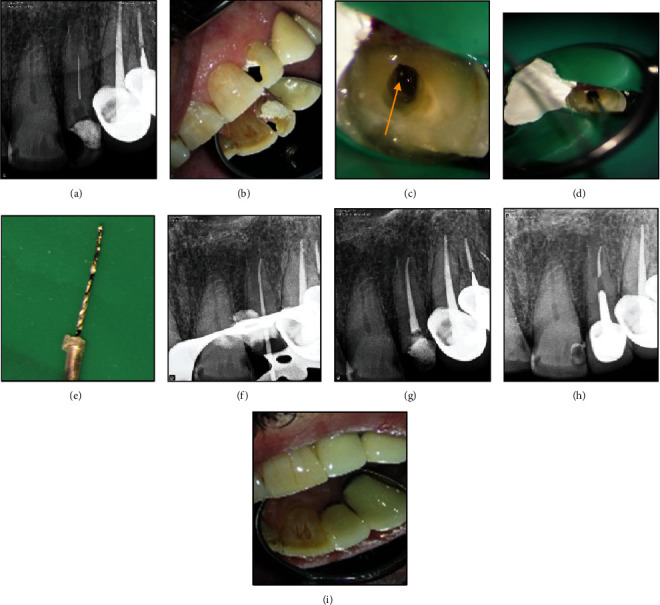
(a) Preoperative radiograph showing a broken instrument in the palatal canal (arrowhead). (b). Intraoral view. (c) Coronal portion of the broken file in the palatal canal was visualized under a DOM. (arrowhead). (d, e) Broken instrument was removed from the canal using the tube-and-glue technique. (f, g) Canal preparation and obturation. (h, i) One-year follow-up: tooth was restored with post and core and metal-ceramic crown.

Enlargement of the coronal third of the canal revealed previous attempts to retrieve the instrument **(**Figure 2(a)**)**. After taking the informed consent, the temporary restoration was removed, and the access cavity was corrected under magnification such that a straight-line access to the coronal portion of the fragment was provided. The dentinal walls around the coronal 2 mm of the broken file were removed by a size 25 U-file on a U-tip (NSK, Nakanishi, Japan) using an ultrasonic instrument (Varios 970; NSK, Japan) under a DOM (Magna Labomed, Labo America Inc., CA, USA) at ×10 magnification. The U-file moved around the broken instrument in a counterclockwise direction. Several unsuccessful attempts were made to remove the broken instrument using the ultrasonic tip. Therefore, the tube-and-glue technique was used to remove the broken instrument. The blunt tip of a 24-gauge needle (Supa, Tehran, Iran) which could encircle the coronal 2 mm of the broken fragment was used as a tube, along with cyanoacrylate glue (Rever Glue, Tehran, Iran). One drop of cyanoacrylate glue was carefully poured on the tip of the tube. The tube was inserted into the canal and held still for 3 minutes to ensure the setting of the glue. With the outward movement of the tube, the broken instrument was successfully removed while entrapped in the tube **(**Figures 2(c), 2(d), and 2(e)**)**. Then, the canals were cleaned with5.25% hypochlorite solution and 17% EDTA, shaped, and obturated with gutta-percha points and AH 26 resin sealer (Dentsply-Tulsa Dental, Johnson City, TN, USA) using the cold lateral compaction technique **(**Figures 2(f) and 2(g)**)**. The patient was then referred for coronal restoration **(**Figures 2(h) and 2(i)**)**.

### 2.3. Case 3: Removal of a Broken Instrument Using the Tube-and-Wire Technique

**Figure 3:**
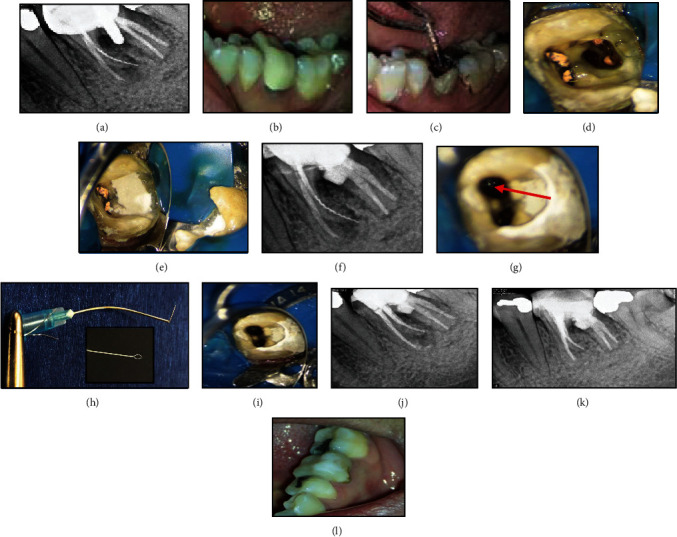
(a) Preoperative radiograph revealing a perforation in the furcation area and a broken instrument in the middle-apical thirds of the mesiolingual root extending beyond the apex. (b) Intraoral view. (c) Removing the post from the distal canal. (d–f) Perforation was sealed using RetroMTA. (g) Coronal portion of the broken file in the palatal canal was visualized under a DOM (arrowhead). (h) The broken file was removed by the tube-and-wire technique. (i) Magnification of the canal shows that the broken file was successfully removed. (j) The canals were prepared and obturated. (k, l) 2-year follow-up: the tooth was restored by composite resin and evidence of healing of the apical and furcal radiolucency was seen.

A systemically healthy 35-year-old female patient was referred to a private endodontic office for assessment of the prognosis of nonsurgical root canal retreatment of the left mandibular first molar. The patient complained of pain on chewing in the aforementioned tooth. Upon clinical examinations, the tooth was slightly sensitive to percussion. The periodontal status of the tooth was normal. A periapical radiograph of the tooth revealed a perforation in the furcation area and a broken instrument in the middle and apical third of the mesiolingual root canal, which was beyond the root apex **(**Figure 3(a)**)**. There was a periapical radiolucency associated with the corresponding root and the furcation area. The patient was informed about the compromised tooth structure and the fair to poor overall prognosis. Following this, informed consent was obtained from the patient. In the first appointment, the porcelain-fused-to-metal crown was cut using a high-speed carbide fissure bur (Dentsply Sirona, Germany). The intracanal post was then removed using a G21 ultrasonic tip (NSK, Nakanishi, Japan) with counterclockwise movement **(**Figure 3(c)**)**. The access cavity was cleaned and irrigated with 5.25% NaOCl. The perforation site was detected under magnification (×10) using a DOM (Magna Labomed, Labo America Inc., CA, USA) and was sealed with RetroMTA (BioMTA, Deajon, Seoul, Korea) **(**Figures 3(d), 3(e), and 3(f)**)**.

Since the quality of the root filling of the distal canals was acceptable, there was no evidence of coronal leakage and apical radiolucency, the previous root canal treatment was done by an endodontist, and selective retreatment of mesial root canals was scheduled. A moist cotton pellet was placed over the RetroMTA to facilitate the proper setting of the cement; the gutta-percha was removed from the mesial canals using ProTaper Universal retreatment file (Dentsply-Maillefer, Ballaigues, Švicarska) with 1200 rpm speed, without using solvent. These files feature end-cutting tips designed to enhance penetration and facilitate the removal of root filling material, thus boosting their efficiency. Additionally, the combination of flute design and recommended usage techniques may also help reduce the risk of file separation. When the coronal portion of the broken file was visualized under the DOM, the dentinal walls surrounding the coronal 2 mm of the broken instrument were circumferentially removed using a size 25 U-file on a U-tip. In the second treatment session, after the temporary filling removal, the setting of RetroMTA was evaluated. A blunt-tip 21-gauge needle (Supa, Tehran, Iran) and a 0.15 mm wire of Tar (string musical instrument) were used. Both ends of the wire were passed through the tube in a way that a lasso was formed **(**Figure 3(g)**)**. Next, the tube was inserted into the canal, and the lasso entrapped the coronal part of the broken instrument. On the other side, the wire was twisted using a needle holder to lock the loop around the fragment. The broken file was successfully removed with some lateral movements and, finally, outward force after three attempts. The canals were then prepared with Neoniti rotary instrument (Neolix, Châtres-la-Forêt, France), irrigated with 5.25% hypochlorite solution and its activation with ultrasonic, and finally, rinsed with 17% EDTA. Subsequently, canals were obturated with gutta-percha points and AH26 resin sealer (Dentsply-Tulsa Dental, Johnson City, TN, USA) using a cold lateral compaction technique **(**Figures 3(i) and 3(j)**)**. The patient was subsequently referred for coronal restoration. At the 2-year recall, the tooth was symptom-free, and the radiolucencies in apical and furcal areas were resolved on periapical radiograph **(**Figures 3(k) and 3(l)**)**.

### 2.4. Case 4: Removal of a Broken Instrument Using BTEX PEN

**Figure 4:**
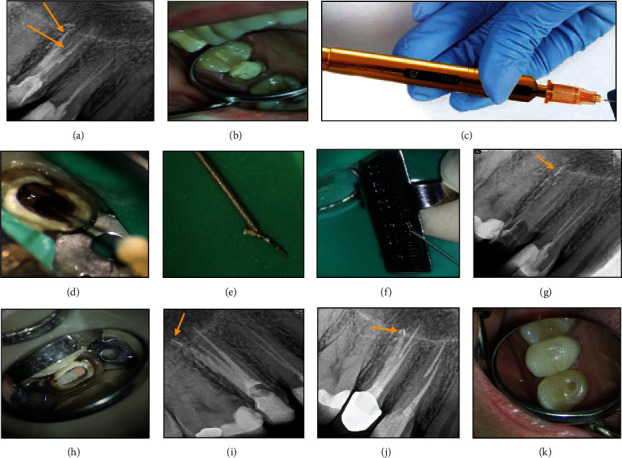
(a) Periapical radiograph revealing a broken instrument in the palatal canal, a broken instrument in the periapical bone, and transportation and perforation in the buccal root (arrowhead). (b) Intraoral view. (c) BTEX PEN. (d–g) Removal of broken file with BTEX PEN. (h) Perforation was sealed with RetroMTA. (i) Canal preparation and obturation. (j, k) 3-year follow-up: the tooth was restored with a metal-ceramic crown and the periapical area appeared to be healthy, with no changes in the surrounding bone on the periapical radiograph.

A 29-year-old healthy female patient was referred to a private endodontic office complaining of pain on chewing on the maxillary right second premolar tooth. The patient reported a history of root canal treatment approximately 4 months earlier. During intraoral examinations, it was observed that the tooth had a faulty restoration and exhibited sensitivity to percussion. However, radiographic examinations revealed that the tooth had a normal periodontal status. Periapical radiograph revealed a broken instrument in the palatal canal, transportation and perforation in the buccal canal, and overextension of an MTA-like material in her previous root canal treatment. Furthermore, there was a separated instrument in the periradicular area (Figure 4(a)). The buccal canal seemed to be filled with an MTA-like material that fortunately was not set and was easily removed by irrigation and ultrasonic agitation. After the removal of guttapercha from the palatal canal, the coronal portion of the broken file was visualized under a DOM (Magna Labomed, Labo America Inc., CA, USA). The coronal 2 mm of the broken file was exposed by a size 25 Ufile on a U-tip using an ultrasonic instrument (Varios 970; NSK, Japan) at ×15 magnification. The U-file moved around the broken instrument with a counterclockwise movement. Since the broken file could not be removed from the canal and further efforts would lead to excessive dentin removal, BTEX PEN (Dimotech, Daya Tajhiz Teb Shomal, Iran), an extractor device, was applied (Figure 4(c)). The broken file was successfully removed after 3 attempts (Figures 4(d), 4(e), 4(f), and 4(g)). A perforation was detected in the buccal canal under a DOM and was sealed with RetroMTA (BioMTA, Deajon, Seoul, Korea) (Figure 4(h)). The canals were then prepared with NiTi rotary instruments (DENCO Super Files III, Longhua, Shenzhen, China), irrigated with 5.25% hypochlorite solution and its activation with ultrasonic, and finally, rinsed with 17% EDTA. Subsequently, canals were filled with EndoSeal MTA (Maruchi, Wonju, Korea) using the single-cone technique (Figure 4(i)). Finally, the patient was referred for coronal restoration. At the 3-year follow-up, the tooth was restored by a metal-ceramic crown. The patient reported no sign or symptom. On the periapical radiograph, the periradicular area seemed to be healthy with no changes in the surrounding bone.

### 2.5. Case 5: Removal of a Broken Instrument Using the Tube-and-Internal Shaft Technique

**Figure 5:**
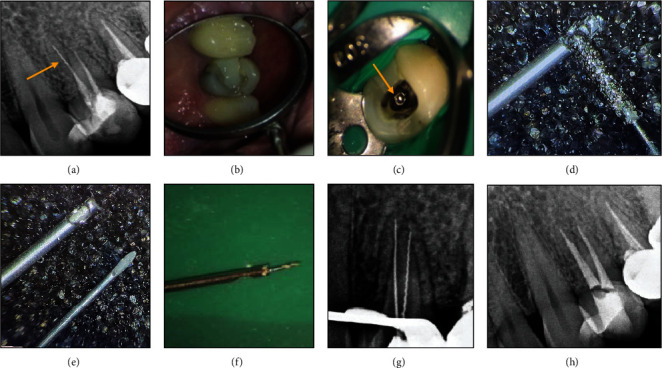
(a) Preoperative radiograph showing a broken file in the apical third of the palatal canal (arrowhead). (b) Intraoral examination showing poor quality composite restoration of maxillary left first premolar. (c) Magnification of the file fragment in the apical third of the canal (arrowhead). (d) A window was created 1 mm away from the tip of the spinal tab needle. (e) Spinal tab needle and internal shaft. (f) Retrieved broken file. (g, h) Canal preparation and obturation.

A 44-year-old healthy female patient was referred to a private endodontic office with a chief complaint of pain while chewing on the maxillary left first premolar. In clinical examination, the maxillary left first premolar had a composite restoration and coronal discoloration (Figure 5(b)). The tooth had a normal periodontal condition. A preoperative periapical radiograph revealed a broken file in the apical third of the palatal canal with no evidence of periapical radiolucency (Figure 5(a)). After the removal of gutta-percha from the canals by NiTi rotary instruments without a solvent, the coronal portion of the broken file was visualized under a DOM (Magna Labomed, Labo America Inc., CA, USA) (Figure 5(c)). The dentinal walls surrounding the coronal 2 mm of the broken fragment were removed by a size 25 U-file on a U-tip using an ultrasonic instrument (Varios 970; NSK, Japan). The sharp tip of a 21-gauge spinal tap needle (Dr. J, Japan) was polished, and a window with a diameter of 1 mm was created 1 mm away from the tip by a fissure diamond bur with a high-speed handpiece without water spray (Figure 5(d)). The coronal 2 mm of the fragment was surrounded by the hollow tube of the spinal tap needle at ×20 magnification. Then, the internal shaft was inserted into the tube in a proper direction in order to push the coronal end of the broken file into the window such that the file would be fixed within the tube. The broken file was successfully removed after two attempts (Figure 5(f)). The canals were prepared with NiTi rotary instruments (DENCO Super Files III, Longhua, Shenzhen, China), irrigated with 5.25% hypochlorite solution and its activation with ultrasonic, and finally, rinsed with 17% EDTA.

Subsequently, canals were obturated with gutta-percha points and AH 26 resin sealer (Dentsply-Tulsa Dental, Johnson City, TN, USA) by the cold lateral compaction technique (Figures 5(g) and 5(h)). The patient was then referred for final restoration.

### 2.6. Case 6: Removal of a Broken Instrument with Stieglitz Forceps

**Figure 6:**
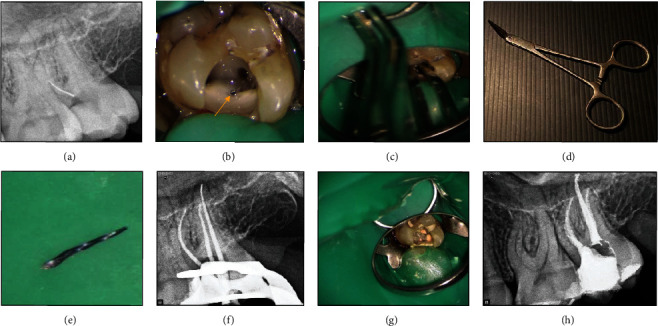
(a, b) A broken instrument in the second mesiobuccal canal (arrowhead). (c–e) Removal of the broken file with Stieglitz forceps. (f–h) Canal preparation and obturation.

A 27-year-old healthy female patient was referred to an endodontist with the chief complaint of pain in her maxillary left second molar. Clinical examination revealed pain on percussion and a normal periodontal status. There was no evidence of periapical radiolucency on the periapical radiograph; however, a broken instrument in the coronal third of the second mesiobuccal canal was detected **(**Figures 6(a) and 6(b)**)**. After the removal of temporary restoration, the broken file in the mesiobuccal canal was visualized under a DOM (Magna Labomed, Labo America Inc., CA, USA). The dentinal walls surrounding the coronal 2 mm of the broken file were removed using an E8D ultrasonic tip (Varios 970; NSK, Japan) at ×10 magnification. Next, the broken file was gripped with Stieglitz forceps (Falcon Medical Italia) and successfully removed from the canal on the first attempt **(**Figures 6(c), 6(d), and 6(e)**)**. The canals were prepared with NiTi rotary instrument (DENCO Super Files III, Longhua, Shenzhen, China), irrigated with 5.25% hypochlorite solution and its activation with ultrasonic, and finally, rinsed with 17% EDTA. Subsequently, canals were obturated with gutta-percha points and AH 26 resin sealer (Dentsply Tulsa Dental, Johnson City, TN, USA) using the cold lateral compaction technique **(**Figures 6(f), 6(g), and 6(h)**)**. The patient was then referred for coronal restoration.

## 3. Search Strategy

The PubMed database (including MEDLINE biomedical literature, life science journals, and online books) was searched for articles published from 2000 to 2023 using the following keywords (MeSH terms): “broken instrument removal,” “ broken instrument retrieval,” “file fracture removal,” “file separation,” and “endodontics.” This search strategy resulted in 325 articles. Fourteen ex vivo and case report studies that applied the methods of broken instrument removal discussed in the present study were then selected and are summarized in Table 1.

## 4. Discussion

Several methods and devices have been proposed for broken instrument removal from the root canal system; nonetheless, no consensus has been reached on a standard method for broken instrument retrieval [15].

To remove the broken instrument from the root canal system, the first step is to establish a straight-line access to the coronal part of the instrument. Next, the dentinal walls surrounding the coronal part of the instrument should be removed in order to expose 1 to 2 mm of the coronal part of the instrument [16]. Although trephine burs have been proposed to facilitate this step, ultrasonic tips are preferred since they outperform other instruments in terms of safe and controlled removal of dentin [17]. In some cases, the broken instrument is solely removed by ultrasonic agitation [12]. In the first case, ultrasonic agitation of the fragment, together with counter-clockwise rotation of the tip around the coronal part of the fragment, led to instrument retrieval.

Based on the results of relevant studies, using ultrasonic alone is time-consuming [18]. Additionally, excessive use of ultrasonic in the apical third of the root canal increases the risk of perforation. On the other hand, excessive efforts to remove a broken file by ultrasonic agitation may lead to excessive radicular dentin removal and the occurrence of iatrogenic errors. Thus, other techniques may be preferred for such cases [19].

The tube-and-glue technique is a less invasive, cost-effective, and accessible method in comparison with other methods. The adhesive properties of the cyanoacrylate glue facilitate bonding to the coronal part of the separated fragment and provide effective retrieval force along with minimal damage to the root canal dentin. However, this technique has limitations; for example, while the glue is setting time, the microtube must be held still around the broken instrument. On the other hand, in some cases, the precise connection between the adhesive and the coronal part of the broken instrument is challenging. The bond between the separated instrument and the glue has to be strong enough to retrieve the fragment; thus, the removal of a large instrument that has been firmly screwed into the radicular dentin may not be feasible with this method [19, 20].

Olczak et al. used three adhesive materials, namely, composite resin, cyanoacrylate glue, and glass ionomer cement for broken file removal by the microtube-and-glue technique. The results showed that a much better connection was obtained by using cyanoacrylate glue than other materials [19]. On the other hand, according to a study by Frota et al., it would be possible to remove a broken fragment from the apical third of the root canal by using this method [20].

In the third and fourth cases, the wire and tube method was used. This method is based on the loop device technique, which was first introduced by Greene [21]. In this method, depending on the diameter of the canal, different gauges of tubes may be used. Also, based on the magnitude of force required to remove the broken instrument, wires with different diameters and tensile strengths may be used. However, one limitation of this method is the absolute need for magnification. Moreover, the skills and experience of the operator have a profound impact on the success of this technique [11].

The BTEX-PEN kit (Dimotech, Daya Tajhiz Teb Shomal, Iran) is a simple broken instrument removal tool based on the loop technique. The kit includes a handle such as a pen with three gauges of needles (25, 27, and 30), and, which are attached to the tip of the handle, and the wires have three diameters of 0.05, 0.08, and 0.1 mm, with different tensile strengths. Based on the canal and broken file size, the appropriate needle gauge and wire diameter should be selected to remove the broken instrument. By twisting the embedded screw on the handle, the radius of the loop is adjusted. In this way, when the loop is placed around the broken piece, the loop can be tightly fixed around the separated fragment. It appears that the time required to remove a broken instrument with this method is less than using an ultrasonic instrument alone [18].

The “tube-and-internal shaft” method is another technique used for broken instrument removal, which was employed in case 5. A spinal tap needle has an internal shaft and is designed in such a way that the internal shaft fits completely inside the needle in only one direction. In this method, initially, a window is created slightly above the tip of the spinal tap needle by a green diamond bur with a high-speed handpiece. The window is created on the same side where the internal shaft sits. When the spinal needle is placed around the broken file, the internal shaft enters the needle from the other side and pushes the broken file into the window. Therefore, the coronal part of the broken file is locked inside the tube and can be removed with a rotational movement [22]. This method, like other methods, requires the use of magnification. Additionally, the operator's experience and skills are highly crucial for the success of this technique.

Based on a study by Alomairy, a lot of time is spent for removing the broken fragments using this method. Moreover, the overall success rate of this method was 60% in his study. Also, in cases where the broken fragment was in the apical third of the root canal, perforation occurred in several instances [22]. This is probably due to the use of a large tube, which requires more dentin removal. By using a tube with a smaller diameter, this issue can be somewhat prevented.

In case 6, the broken instrument was firmly grasped by the Stieglitz forceps and easily removed from the root canal. This method is applicable in instances where the broken instrument is located in the coronal third of the canal. This method can be especially used to remove the broken pins and remaining silver cones from the root canal. One limitation of this method is that fragments that are deeply situated in the canal cannot be grasped and removed by this approach [23].

One of the most important advantages of tube-based techniques in this study is their compatibility with affordable and easily accessible equipment. This eliminates the necessity of relying on costly commercial kits. However, the time required for the preparation of these techniques might surpass that of utilizing a ready-made kit for the removal of broken instruments, which poses a disadvantage.

By evaluation of the data retrieved from the reviewed studies presented in Table 1, the following conclusions may be drawn:

- Removal of broken instruments using an ultrasonic instrument alone is more time-consuming than other methods
- The risk of iatrogenic incidents such as perforation is higher when the broken fragment is in the apical third of the root canal, compared to the middle or coronal third
- In the internal shaft method, significant removal of dentin is often required due to the large diameter of the tube, which in turn increases the risk of root perforation
- Forceps-based methods are generally effective in the coronal third of the root canal.
- Type and size of the instrument, whether hand instrument or rotary instrument, do not appear to have an impact on the success of broken file removal

Generally, the following instructions are recommended for the nonsurgical method of the broken instrument retrieval, taking into account the merits and demerits of each approach and various clinical circumstances, including the broken instrument size and location, canal curvature, and canal diameter:

1. The utilization of the dental operating microscope (DOM) along with ultrasonic tips is one of the most important components for minimally invasive root canal preparations and facilitates the safe removal of fractured instruments
2. Using small-diameter ultrasonic tips alone has the potential to efficiently and quickly remove small and coronally broken fragments. If the use of ultrasonic to remove a fractured fragment takes longer than expected and does not achieve the desired result, there is an increased risk of iatrogenic events such as excessive dentin removal, ledge, transportation, and perforation or even second fracturing of the ultrasonic tip. In these situations, it is important to consider alternative techniques for instrument removal
3. Fractured instruments located in the coronal third of the root canal can be readily removed using forceps-based techniques, provided there is sufficient space around the broken fragment. This method is a convenient approach for the removal of such instruments
4. The tube and internal shaft technique is applicable for smaller fractured instruments in anterior teeth or straight portions of the root canals. However, it is not appropriate for larger fragments found in the curved canal or in narrow root canals
5. If the use of ultrasonic tips proves ineffective in removing broken instruments, such as large and apical fragments in curved canals, alternative techniques such as the “tube and glue” and “tube and wire” methods can be employed to successfully and conservatively extract the broken instrument

## Figures and Tables

**Table 1:** Summary of the five techniques of broken instrument removal in case reports and ex vivo studies.

	Article	Type of teeth	Instrument type	Instrument length	Location in root	Time or attempt	Success	Perforation
Ultrasonic	Souter and Messer [11] Ex vivo	Mandibular molars	Profile file (#35/.04 taper)	3 mm	Apical third 15	N/A	11 from 15	3
	Souter and Messer [11] Ex vivo	Mandibular molars	Profile file (#35/.04 taper)	3 mm	Coronal third 14	N/A	100%	N/A
	Souter and Messer [11] Ex vivo	Mandibular molars	Profile file (#35/.04 taper)	3 mm	Middle third 16	N/A	100%	N/A
	Souter and Messer [11] Clinical study	Mandibular molars	N/A	N/A	Apical third	N/A	9 from 27	7
	Souter and Messer [11] Clinical study	Mandibular molars	N/A	N/A	Coronal third 11	N/A	100%	N/A
	Souter and Messer [11] Clinical study	Mandibular molars	N/A	N/A	Middle third	N/A	100%	N/A
	Shahabinejad et al. [24] Ex vivo	Maxillary premolars	#30/.04 taper Hero file	3 m	Middle third 8	32.62 min	N/A	N/A
	Shahabinejad et al. [24] Ex vivo	Maxillary premolars	#30/.04 taper Hero file	3 mm	Apical third 27	37.48 min	N/A	N/A
	Alomairy [22] Ex vivo	First and second molars	Profile rotary Ni-Ti 25 6%	4-mm	Apical	55 min	80% 12 from 15	1 from 15
	Pruthi et al. [25] Ex vivo	First mandibular molars	Pro taper F1 rotary instruments	4 mm	coronal third	17.9minutes	100%	N/A
	Pruthi et al. [25] Ex vivo	First mandibular molars	ProTaper F1 rotary instruments	4 mm	Middle third	46.4 minutes	80% 16/20	N/A

Tube -and-glue	Frota et al. [20] Case report	Maxillary left first premolar	Hand file	6 mm	apical third	5 minutes	N/A	N/A
	Andrabi et al. [26] Case report	Mandibular first molar	ProTaper shaper SX	N/O	Coronal third	N/A	N/A	N/A

Tube -and- wire	Terauchi et al. [27] Case report	Mandibular second molar	N/A	5 mm	Apical third	7 min	N/A	N/A
	Terauchi et al. [27] Case report	Mandibular second molar	K file	5 mm	Apical third	5 min	N/A	N/A
	Terauchi et al. [27] Case report	Mandibular third molar	K file	8 mm	Apical third	6 min	N/A	N/A
	Terauchi et al. [27] Case report	Mandibular first molar	NiTi instrument	4 mm	Apical third	12 minutes	N/A	N/A
	Terauchi et al. [9] Case report	Mandibular first molar	K file	14 mm	Coronal to apical	31 min	N/A	N/A
	Pruthi et al. [25] Ex vivo	First mandibular molars	ProTaper F1 rotary instruments	4 mm	Coronal third	15.3 minutes	100%	N/A
	Pruthi et al. [25] Ex vivo	First Mandibular molars	ProTaper F1 rotary instruments	4 mm	Middle third	44.2 minutes	90% 18/20	N/A

Tube -and- Internal shaft	Yang et al. [18] Case report	Mandibular intact molars	K3 file size 25/.06	4 mm	Apical third	8.9 min	N/A	N/A
	Cruz et al. [28] Case report	Mandibular first molar	15 or 20 size Hand instrument	N/A	Middle to apical beyond the apical foramen	N/A	N/A	N/A
	Alomairy [22]	First and second molars	Profile rotary Ni-Ti 25 6%	4 mm	apical	55 min	60% 9 from 15	3 from 15
	AlRahabi and Ghabbani [29] Case report	Maxillary canine	NiTi rotary (F3 of the ProTaper	16 mm	Coronal to apical	Three attempts	N/A	N/A
	Al-Zahrani and Al-Nazhan [30] Case report	Maxillary lateral incisor	Profile sizes: 35 and 25	N/A	Middle to apical	First attempt	N/A	N/A

Forceps	Ahmed et al. [31] Case report	Upper lateral incisor	Hand instrument	5 mm	Coronal	Easy to remove	N/A	N/A
	Meidyawati et al. [32] Case report	Mandibular first molar	N/A	N/A	Coronal	N/A	N/A	N/A

## Data Availability

The data supporting the findings of the present study will be provided by the corresponding author upon request.
